# A detailed methodology for estimating health-related hazards of workplace exposure to indoor formaldehyde vapours

**DOI:** 10.1016/j.mex.2024.102937

**Published:** 2024-08-28

**Authors:** Marzieh Belji Kangarlou, Alireza Dehdashti, Elaheh Saleh

**Affiliations:** a Student Research Committee, Semnan University of Medical Sciences, Semnan, Iran; b Department of Occupational Health, Faculty of Medical Sciences, Tarbiat Modarres University, Tehran, Iran; c Social Determinants of Health Research Center, Semnan University of Medical Sciences and Health Services, Semnan, Iran; d Social Determinants of Health Research Center, Semnan University of Medical Sciences, Semnan, Iran; e Department of Public Health, Damghan school of Health, Semnan University of Medical Sciences, Semnan, Iran

**Keywords:** Occupational exposure, Environmental monitoring, Biological monitoring, Respiratory symptoms, Risk assessment, Application of Biological, carcinogenic and non-carcinogenic health risk assessment of formaldehyde in hospital laboratory

## Abstract

A comprehensive risk assessment method was applied to examine the risks associated with airborne formaldehyde occupational exposure among hospital laboratory staff. The method assessed exposure levels and health impacts by integrating area and personal air sampling, biological monitoring, and self-reported health data. Samples were collected from 74 workplaces across various departments using NIOSH method 3500 and were analyzed via UV-vis spectrophotometry. The data showed significant differences in exposure levels between departments (p≤0.05) and confirmed the efficacy of the method in identifying risk differences. Despite average personal exposure levels being measured lower than occupational limits, individual assessments indicated that some participants surpassed these limits, emphasizing the necessity of personal monitoring for workers with higher risks. The high prevalence of respiratory symptoms, such as cough and wheezing among staff, indicated the need for further investigation and targeted interventions. Although estimated cancer and non-cancer risks were within safe thresholds, the study emphasized the importance of continuous exposure monitoring and the implementation of effective control measures in hospital laboratory departments with formaldehyde emission. This integrated method improved the reliability and generalizability of formaldehyde exposure risk assessments and aided in the development of safe occupational health practices.

• The method integrated personal and area sampling with advanced calibration for precise occupational exposure evaluation in laboratories.
• The method used of biomarkers to assess formaldehyde absorption in the body estimating both cancerous and non-cancerous health risks associated with occupational exposure.
• Addressed traditional method limitations and integrated risk components to improve data reliability for workplace safety and health risk management.

## Graphical abstract

**Figure:**
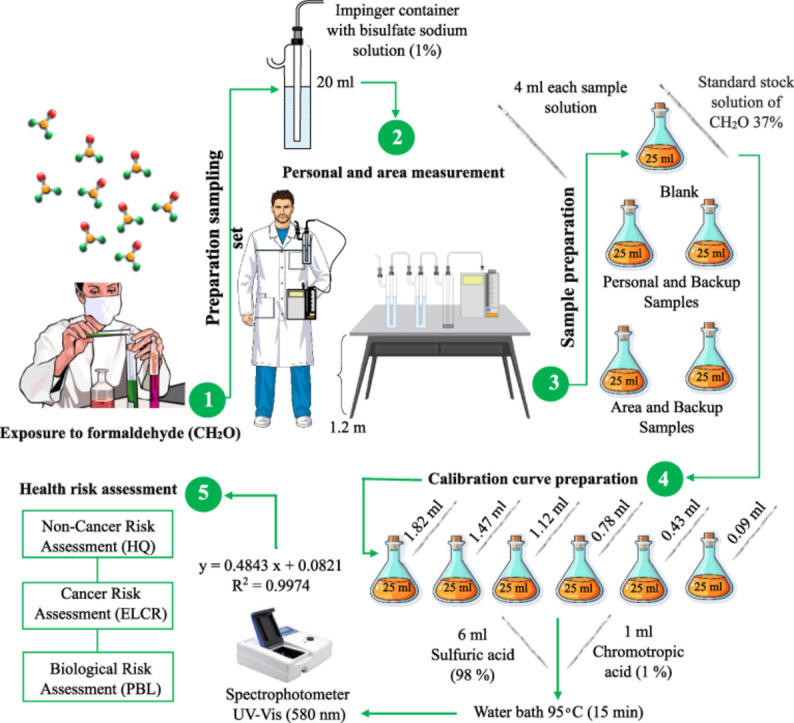

Specifications table

**Table:** 

Subject area:	Environmental Science
More specific subject area:	Health risk assessment of formaldehyde in hospital laboratory
Name of your method:	Application of Biological, carcinogenic and non-carcinogenic health risk assessment of formaldehyde in hospital laboratory
Name and reference of original method:	Occupational health risk assessment of airborne formaldehyde in medical laboratories, Environmental Science and Pollution Research, 30 (17) 2023, 50392-50401
Resource availability:	The data are available with this article.

## Background

### Problem statement

Formaldehyde is known as a carcinogen in workplaces and is commonly used in hospitals for germicides, preserving tissues and fixing specimens. There are concerns about potential health risks associated with work environment exposure. Occupational exposure to formaldehyde has been linked to upper respiratory tract irritation, including burning eyes, cough, and wheezing [1] and also increased risk of nasopharyngeal cancer, leukemia, and other malignancies [2]. Thus, the presence of formaldehyde in medical laboratories thus constitutes a considerable occupational hazard. A comprehensive risk assessment of formaldehyde health hazards should involve measuring and evaluating occupational exposure to airborne concentrations., estimating the entry and absorption of formaldehyde in the body, estimating the potential cancerous and non-cancerous effects on workers, and evaluating respiratory health symptoms in exposed workers. The assessment is complemented by a quantification of risk factors, which involves determining the probability of occurrence and the severity of outcomes on health [3].

These potential health hazards make it crucial to understand and mitigate the risks associated with formaldehyde exposure in workplace environments particularly in hospital laboratories where its use is prevalent. Health hazards and safety risk assessment of formaldehyde in work settings are crucial for the working population and industries due to the significant health risks associated with formaldehyde exposure. Implementing risk assessment protocols, such as identifying exposure levels and implementing corrective actions, is essential to reduce formaldehyde exposure and protect the health of workers in different occupational settings [4]. By evaluating biomarkers and exposure levels, industries can mitigate risks, improve workplace safety, and safeguard the well-being of employees exposed to formaldehyde [5].

### Current ongoing solutions

Despite these potential health risks, some limitations hindered a comprehensive understanding of these risks. Current methods for assessing formaldehyde exposure primarily rely on area sampling techniques, which include measuring formaldehyde air concentrations in different locations within the laboratory environment [6]. While useful in determining how air movement and sources contribute to airborne concentrations, these methods have critical limitations. They cannot show the variability in individual exposures among laboratory personnel, leading to potential underestimations of risk [7]. Limited exposure assessment by solely relying on area sampling might not capture individual exposure variations, potentially underestimating risks for staff handling formaldehyde solutions [3,8]. These assessments typically involve identifying formaldehyde sources, measuring exposure levels, and estimating the risk of adverse health effects [9]. However, many existing approaches do not incorporate biological monitoring which is essential for understanding the internal dose and its potential health impacts on workers.

### The proposed methodology in this work study

Our proposed solution involves a comprehensive risk assessment framework that integrates personal and area sampling with biological monitoring and health risk assessments. This method article describes a detailed multidimensional method to assessing occupational exposure to formaldehyde. It incorporates both personal and area sampling to account for individual behavioral variations and exposure patterns, enhancing the reliability and generalizability of the risk assessment results [10]. By estimating biological absorbed doses and peak blood levels of formaldehyde, the method provides valuable insights into the internal exposure burden among laboratory staff. The inclusion of a standardized respiratory symptoms questionnaire further helps in evaluating the potential health effects associated with formaldehyde exposure.

### Current research novelty and objectives of this work

To conduct a comprehensive health hazards risk assessment method for occupational exposure to formaldehyde vapor in medical laboratory settings, the method addressed the research gaps in indoor area and personal exposure measurements, the limited use of biomarkers, and the estimation of cancerous and non-cancer risks associated with formaldehyde exposure [11]. This comprehensive risk assessment framework not only addressed the limitations of previous studies but also set a new standard for evaluating and managing formaldehyde exposure in medical laboratory settings.

The significance of this methodological study extends beyond the limits of medical laboratories. The healthcare industry, regulatory bodies, and occupational health professionals can benefit from a standardized and reliable method for assessing chemical exposures. The primary objective of this work was to enhance the health and safety of laboratory personnel by providing detailed data on exposure levels and health risks.

For society, this research highlights the potential health and safety risks associated with formaldehyde exposure and emphasizes the need for effective monitoring and risk management practices. From an industry perspective, the findings and methodologies presented in this study offer valuable insights into best practices for formaldehyde use and management in medical laboratories.

## Method details

### Participants

This risk assessment study involved a total of 133 staff members working in hospital medical laboratories. Participants were recruited from 74 laboratory workplaces across the participating hospital. Inclusion criteria ensured that participants were willing to participate in the study and had at least one year of experience working in laboratory activities that utilized formaldehyde. The minimum one-year work experience criterion aimed to include participants who had sufficient exposure duration to potentially experience health effects. The inclusion criteria focused on capturing data from individuals directly involved in laboratory activities that use formaldehyde, ensuring relevance to the study's objectives. Individuals with a history of pre-existing respiratory disorders diagnosed before their employment in the laboratory were excluded. This helped to isolate the potential effects of formaldehyde exposure in the workplace and avoid confounding factors.

Furthermore, to isolate the effects of formaldehyde exposure and minimize confounding factors, individuals with a history of smoking or known allergies to formaldehyde or other chemicals commonly used in the laboratories were excluded from the study. Of all participants, 24 did not meet the inclusion criteria most likely due to lack of the required one-year work experience with formaldehyde.

### Method of sampling and analysis

NIOSH method number 3500 was applied for collecting air samples [6].

### Sampling types

Area and personal air samples were collected from 74 workplaces in the hospital laboratory department to measure airborne formaldehyde concentration resulting in a collection of 185 air samples. Additionally, personal air sampling was conducted for staff members in the laboratory departments to measure individual exposure levels. Area integrated monitoring sampler was positioned in a stationary location at a height of about 1.2 meter from the floor to evaluate the level of formaldehyde vapor in laboratory rooms. An integrated monitoring sampler was connected to the worker to conduct personal samplings from the breathing zone of selected laboratories staff. personal samplers is placed on the employee to determine individual exposures to account for employee mobility issues in obtaining accurate and representative exposure samples [12].

### Sampling duration

The sampling was conducted during normal working hours (8:00 a.m. to 4:00 p.m.).

### Sampling equipment and calibration

The SKC Air Check PCXR4 sampling pump was used for this study. The personal sampling pump was calibrated by soap bubble flow meter before sampling.

### Sampling procedure

Two glass midget impingers containing 20 ml of 1 % sodium bisulfate solution and a trap bottle were serially connected to each other and consequently were set to a sampling pump at a flow rate of 1 l/min. The working range for this method was 0.02 to 4 ppm (0.025 to 4.6 mg/m3) for an 80-L air sample.

### Sampling locations

The sampling locations were selected in the laboratory ambient where formaldehyde vapors were expected to be present. Air samples were collected from various hospital laboratory workspaces located at the divisions of pathology, bacteriology, hematology, biochemistry, blood bank, serology, reception and services rooms.

### Sample preparation

4-ml aliquots from each sample solution were pipetted into 25-ml glass-stoppered flask. Then, 1 ml of chromotropic acid 1 % and 6 ml of sulfuric acid 98 % were added to each sample. Then, the samples were kept in water bath at 95°C for 15 min.

### Calibration curve preparation

Standard stock solution of formaldehyde was prepared from 37 % formalin solution at a concentration of 0.91 mg/ml. Then, standard solutions were made at concentrations of 0.09-1.82 µg/ml by diluting standard solution using 1 % sodium bisulfite. the absorbance was read using the UV-Vis spectrophotometer at 580 nm (absorbance vs micrograms of formaldehyde per milliliter). Additionally, the daily calibration was done with the working standards.

### Formaldehyde concentration determination

The concentration of formaldehyde in samples was calculated by comparing to the absorbance of standard samples. The samples were analyzed using visible absorption spectrometry. The chromotropic acid and sulfuric acid method was used for color development. The absorbance was measured at 580 nm.

### Quality control

**Fig. 1:**
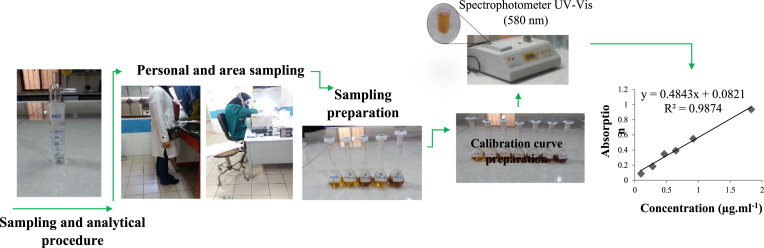
Integrated sampling and analytical setup and implementation for assessing formaldehyde exposure in medical laboratories.

Field blanks were used during air sampling to assess background contamination. Analytical instruments were calibrated using standard procedures, and detection limits were established to ensure reliable formaldehyde concentration measurements. Data integrity was maintained through proper record-keeping and chain-of-custody protocols. The timeline of study design is shown in Fig. 1

### Estimating biological absorbed dose and peak blood level of formaldehyde

The amount of formaldehyde absorbed into the body through the lungs was estimated by measuring the levels of formaldehyde in blood samples [13,14]. The peak blood level (PBL) of formaldehyde was estimated in individuals exposed to formaldehyde vapors in hospital laboratories.

The amount of formaldehyde absorbed into the body through the lungs was estimated using the following formula:

Biological Absorbed Dose BAD (mg) = C (mg/m³) × T (hr) × V (m³/hr) × R ( %)

C: Concentration of formaldehyde vapor in inhaled air (mg/m³) refers to the measured concentration of the target formaldehyde in the inhaled air, typically expressed in milligrams per cubic meter (mg/m^3^). The concentration data is obtained through air sampling near the worker's breathing zone.

T: Duration of participants' exposure (hours) representing the length of time a worker is exposed to the contaminant, usually expressed in hours. Exposure duration can be obtained from monitoring data (e.g., time spent wearing a personal sampling device) or through work shift schedules.

V: Pulmonary ventilation rate (assumed as 1.2 m³/hr for adults at rest) reflecting the volume of air inhaled and exhaled per unit time during respiration. In your formula, a ventilation rate of 1.2 m^3^/hr is assumed. This value represents a typical resting adult breathing rate and is a common assumption for occupational exposure scenarios.

R: Average respiratory retention, representing the fraction of inhaled formaldehyde absorbed and not exhaled. Previous study reported a value of 98 % indicating near-complete retention of formaldehyde in the lungs based on model [15].

Limitation:

The Biological Absorbed Dose was estimated using a standard formula, considering a pulmonary ventilation rate of 1.2 m^3^/hr and a respiratory retention rate of 98 % represent common assumptions but might not account for individual variations or specific activities that could influence inhalation rates. The retention rate is influenced by factors like the contaminant's vapor pressure, solubility in blood, and interaction with lung tissue. a portion might be exhaled unchanged, metabolized in the lungs, or deposited and slowly released from deeper tissues [16]. While these values represent typical assumptions for occupational settings, future studies could explore activity-specific adjustments or personalized physiological data for more refined BAD estimates.

The peak blood level (PBL) of formaldehyde in individuals exposed to formaldehyde vapors in hospital laboratories is estimated by dividing the calculated BAD by the volume of body fluid [17]:

PBL (mg/L) = BAD (mg) / Body Fluid Volume (L)

Body Fluid Volume was considered as the volume of blood in a standard 70 kg adult (assumed as 42 L, accounting for 60 % water content).

Limitations:

Individual variability: Body fluid volume and other physiological factors can vary significantly between individuals, affecting PBL estimations.

Static model: This method assumes constant exposure and absorption throughout the exposure period, which may not be realistic in all cases.

### Risk characterization

Occupational exposure to formaldehyde has been linked to an increased risk of cancer, particularly of the nasal sinuses and nasopharynx. This study estimated the excessive lifetime cancer risk (ELCR) from inhaled formaldehyde involved calculating the inhalation unit risk (IUR) of formaldehyde, which is the increased risk of developing cancer due to exposure to a specific level of formaldehyde over a lifetime [18,19]. Excessive Lifetime Cancer Risk (ELCR) from inhaled formaldehyde was calculated using the following equation:

ELCR = EC × IUR × EF × ED / AT

where:

- EC (µg/m^3^): Time-weighted average concentration of formaldehyde based on sampling data and occupational exposure limits.
- IUR ((µg/m^3^)-1): Inhalation Unit Risk for formaldehyde obtained from reputable sources (e.g., USEPA).

The Inhalation Unit Risk (IUR) of Formaldehyde used in our assessment was 1.3 × 10^-5^ per µg/m³ obtained from the USEPA IRIS database [20]. This means that for every 1 microgram of formaldehyde inhaled per cubic meter of air, there is a 1.3 × 10^-5^ increased risk of developing cancer. The IUR for formaldehyde is based on prior studies that have shown an increased risk of respiratory cancers, including nasal cavity and paranasal sinus cancers, nasopharyngeal cancer, and lung cancer [21].

- EF (days/year): Exposure frequency (e.g., number of workdays per year).
- ED (years): Exposure duration (number of working years).
- AT (days): Average lifetime (years x 365 days/year).

Limitation:

IUR describes a conventional estimate and may not reflect the full spectrum of susceptibility within the population. However, it provides a valuable tool for risk assessment in occupational settings.

### Hazard quotient (HQ)

The hazard quotient (HQ) assessed the potential for non-cancer health risks from exposure to a single chemical [22]. It compares the estimated exposure level to a reference concentration (RfC) established by regulatory agencies. The Hazard Quotient (HQ) was calculated by dividing the time-weighted average concentration of formaldehyde (EC) measured in the workplace air by the draft RfC value. If the HQ is less than 1, it suggests that the exposure level is unlikely to cause adverse health effects. HQ was calculated using the equation:

HQ = EC / RfC

where:

- EC (µg/m^3^): As defined previously for ELCR calculation.
- RfC (µg/m^3^): Inhalation Reference Concentration for formaldehyde

A draft RfC value of 3.94 × 10⁻⁵ µg/m³ was used, based on sensory eye irritation from reference concentrations for non-cancer effects as reported by the Environmental Protection Agency's draft IRIS assessment of formaldehyde. [23]. This health effect was chosen as the basis for the RfC due to its established association with chronic inhalation exposure to formaldehyde.

### Respiratory symptoms questionnaire

To assess potential respiratory health effects from airborne formaldehyde exposure, all participants completed two validated respiratory symptom questionnaires: the American Thoracic Society Questionnaire (ATSQ) and the Health and Safety Executive/Health and Safety Laboratory (HSE/HSL) questionnaire (validity established in [24,25]). These questionnaires explored the frequency of various respiratory symptoms commonly associated with formaldehyde inhalation. This data on self-reported symptoms complements the objective airborne formaldehyde concentration measurements, aiding in the identification of potential exposure effects.

### Pulmonary function tests

To objectively assess lung function and identify potential respiratory impairments, spirometry was employed as the primary tool for pulmonary function testing (PFTs). A spirometer was used to measure the volume and flow rate of air inhaled and exhaled during forced maneuvers. All participants underwent standardized pulmonary function testing procedures following established guidelines [26]. These procedures involved taking deep breaths and forcefully exhaling into the spirometer mouthpiece. The spirometer measurements of various lung function parameters included:Forced Vital Capacity (FVC): The total volume of air forcefully exhaled after a maximal inhalation. Forced Expiratory Volume in 1 second (FEV_1_): The amount of air forcefully exhaled in the first second of a forced exhalation. Peak Expiratory Flow Rate (PEFR): The maximum rate of airflow during forced exhalation. Forced Expiratory Flow 25-75 % (FEF _25-75 %_): The average flow rate during the middle half of a forced exhalation. The obtained values were compared with reference ranges adjusted for age, height, and sex, to identify participants with potential lung function abnormalities.

### Statistical analysis

#### Data normality test

The normality of data distribution for airborne formaldehyde concentrations (personal and area measurements) was assessed using the Kolmogorov-Smirnov test with SPSS software.

#### Exposure level comparison

A one-sample t-test was performed to compare the mean airborne formaldehyde concentration measured through personal sampling with the occupational exposure limit. The Kruskal-Walli's test was conducted to compare the means of personal and area formaldehyde concentrations across different laboratory departments.

#### Significance level

The level of statistical significance was set at *p* ≤ 0.05.

## Method validation

### Significant differences between personal and area exposure

**Fig. 2:**
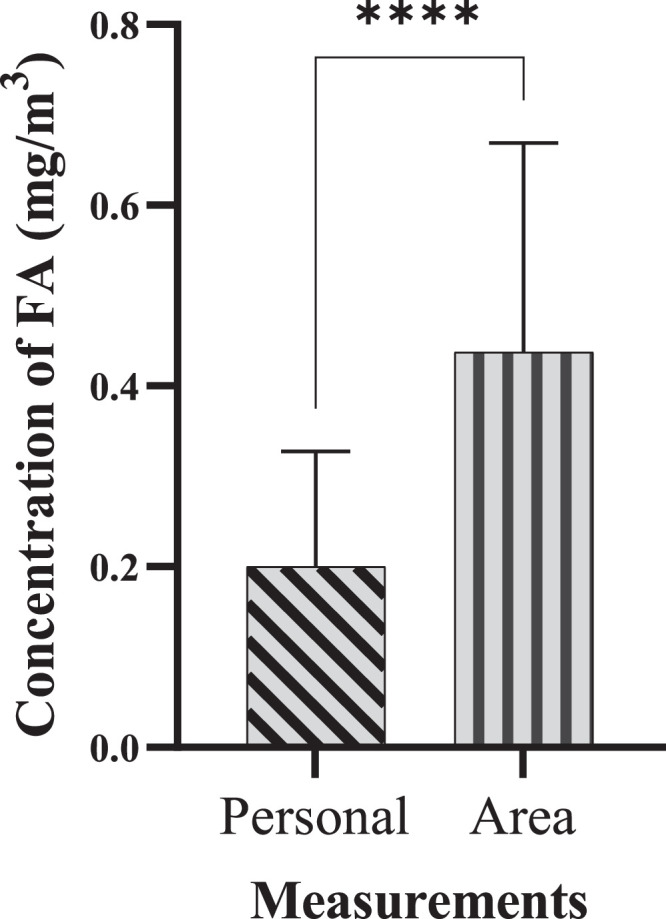
Comparison of personal and area exposure to airborne formaldehyde in laboratory workspaces (****P-Value ≤ 0.0001).

The study data demonstrated a statistically significant difference (P-value ≤ 0.001) between personal and area airborne formaldehyde exposure levels for most laboratory departments (Fig. 2). This validates personal sampling approach, as it directly captures the formaldehyde concentration within an individual's breathing zone, potentially reflecting their specific tasks and proximity to sources which may differ from general air concentrations measured through area sampling.

Both area and personal measurements were performed to determine occupational exposure data. Area measurements of airborne contaminants provided data on the contribution and role of sources and air movement in generating airborne formaldehyde throughout the work environment. Personal sampling and measurements produced data concerning the workers' behaviours at work and their links to contaminant sources that may affect their exposure levels. while area measurements might indicate elevated formaldehyde levels near a specific piece of equipment, personal measurements can reveal that workers who frequently use this equipment or work in its vicinity have higher exposure levels than those in other areas. This differentiation is critical for implementing targeted interventions and improving workplace safety.

The combination of personal and area measurements improved the method's ability to identify potential risk areas by providing comprehensive exposure data compared to using either method alone [27]. Personal measurements allowed for a detailed assessment of individual exposure capturing the variations due to specific tasks and workers’ interaction with sources. Meanwhile, area measurements provided an overall exposure data of the spatial distribution of formaldehyde, identifying zones within the workspace that might require targeted interventions.

In our risk assessment of the workplaces, high formaldehyde levels detected in both area and personal measurements indicated critical risk areas requiring immediate intervention. In situations where area measurements show high levels but personal measurements do not, it suggested safe work practices that might mitigate exposure.

### Exposure variations across departments and mitigation

The data revealed variations in formaldehyde exposure across different hospital laboratory departments where formaldehyde use might be more prominent. This highlights the effectiveness of the methods in detecting variations in potential risks depending on specific laboratory activities. The highest exposures based on personal and area airborne formaldehyde were found in bacteriology, pathology, and services departments, respectively potentially due to the lack of effective local ventilation and, the use of formalin fixatives or other formaldehyde-containing solutions. The data suggest that formaldehyde levels in most departments exceeded the recommended exposure limits indicating that the method can be used to evaluate adherence to safety standards. Moreover, the one-samples t-test comparing the mean scores of area and personal airborne formaldehyde measurements with recommended exposure limits all sampling sites found a significant difference between the means of the two measurements except pathology department that this significant relationship was not observed (P-value=0.423, 0.409). This indicated the method's effectiveness in detecting departures from safe exposure limits.

**Fig. 3:**
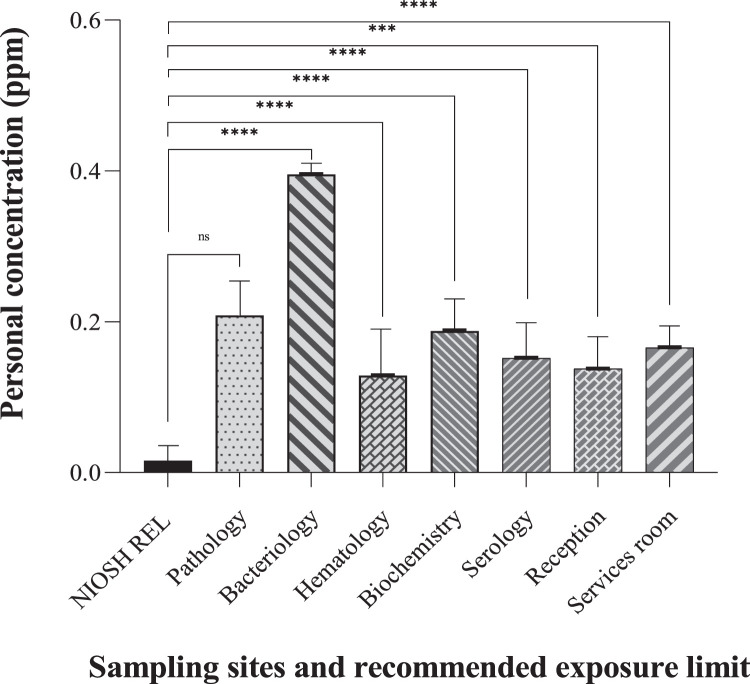
Personal exposure of formaldehyde (ppm) in various sampling sites of laboratory workplaces and comparison with recommended exposure limit (****P-Value ≤ 0.0001).

**Fig. 4:**
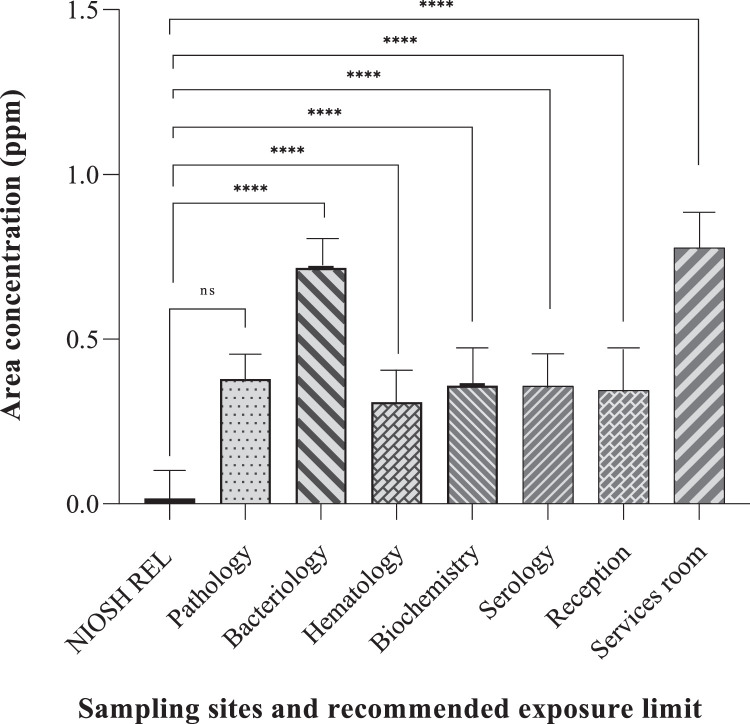
Area airborne concentration of formaldehyde (ppm) in various sampling sites of laboratory workplaces and comparison with recommended exposure limit (****P-Value *≤* 0.0001).

Personal measurements provided data results about individual's potential exposure while area measurements pointed out a general idea of airborne formaldehyde levels within a particular workspace. This comprehensive approach strengthened the method's ability to identify potential risk areas. By combining personal and area measurements and comparing them to a standard limit, the method offered valuable data. However, future studies with a larger sample size and potentially incorporating personal monitoring over a longer duration could provide an even more robust validation of the method (Fig. 3, Fig. 4).

### Comparison with recommended exposure limits

**Fig. 5:**
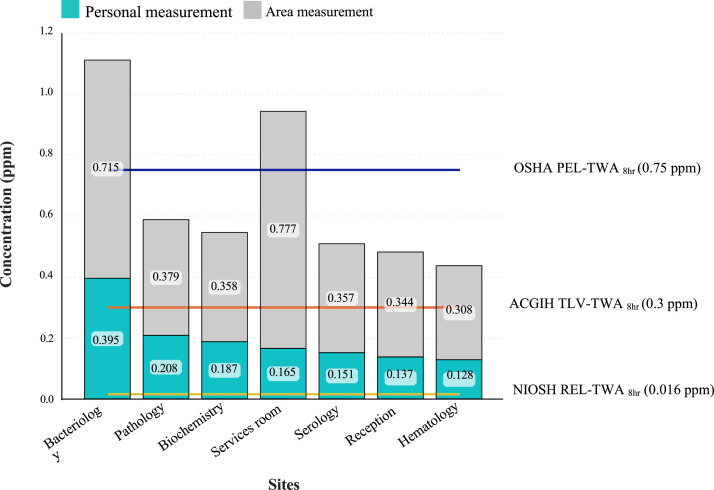
Personal and area concentrations of formaldehyde (ppm) in various sampling sites of laboratory workplaces and comparison with occupational exposure limits.

While the mean personal exposure levels were below the OSHA Permissible Exposure Limit (PEL) of 0.75 ppm, individual exposures exceeded OEL in 20 % of the participants (Fig. 5). This highlights the importance of considering various regulatory standards and the specific context when evaluating potential health risks.

The data presented in Fig. 5 validates our method by highlighting the critical differences between personal and area formaldehyde concentrations across various laboratory settings. While average personal exposure levels are below OSHA's PEL of 0.75 ppm, individual exposures exceeded this limit in 20 % of measurements, particularly in high-risk areas like bacteriology and pathology. By benchmarking against NIOSH REL (0.016 ppm) and ACGIH TLV (0.3 ppm), our method ensured worker safety evaluation across multiple regulatory standards [28]. Furthermore, these data emphasized the need for enhanced ventilation and targeted risk mitigation strategies.

Factors contributing to elevated exposure in pathology include high usage of formaldehyde, inadequate ventilation, suboptimal work practices, and improper storage and disposal of formaldehyde-containing materials. Installing Local Exhaust Ventilation (LEV) systems such as fume hoods and downdraft tables at workstations may reduce formaldehyde exposure. The pathology department can apply ventilated enclosures for specimen handling, while the services department should utilize fume cupboards during formaldehyde preparation. General ventilation by increasing air changes per hour can also reduce exposure to formaldehyde vapours and improve air quality. Designing automated and closed system processing, provision of appropriate personal protective equipment (PPE), and regular training and education for employees may mitigate the risks. Also, routine exposure monitoring and health surveillance are essential to ensure compliance with occupational health and safety standards and early detection of overexposure [27].

### Estimated biological levels

The estimated peak blood levels (PBL) of formaldehyde based on both personal and area exposure were generally low across most departments, with the exception of the pathology department. The combined approach of measuring biological exposure on the basis of personal and area exposure provides a more comprehensive picture of potential risks. This allows for identifying high-risk areas such as pathology and bacteriology, where further investigation and control measures might be necessary. Additionally, the calculation of PBL provides insights into the potential amount of formaldehyde absorbed by individuals. This offers a personalized risk assessment approach compared to exclusively relying on area concentration measurements.

The data reveals significant variations in exposure levels between different laboratory sections. Pathology and bacteriology departments, as expected, exhibited the highest exposure levels, with some exceeding the OSHA occupational exposure limit for PBL. This indicates the importance of targeted assessments and potential risk mitigation strategies in these specific areas (Table 1, Table 2).

**Table 1:** Risk calculations of health hazard assessment based on personal formaldehyde exposure in laboratory workplaces.

Personal Risk Assessment		Sampling Sites						
		Pathology	Bacteriology	Hematology	Biochemistry	Serology	Reception	Services room
EC _noncancer_ (µg/m3)	Max	0.0956	0.002398	0.000782	0.001144	0.000922	0.000836	0.001005
	Mean	0.0647	0.002400	0.000800	0.001100	0.000900	0.000800	0.001000
	Min	0.0036	0.002394	0.000778	0.001132	0.000916	0.000832	0.001001
HQ	Max	0.0913	0.0001	0.0198	0.0290	0.0233	0.0212	0.0255
	Mean	0.0321	0.0610	0.0200	0.0290	0.0230	0.0210	0.0250
	Min	0.0028	0.0003	0.0197	0.0287	0.0232	0.0211	0.0254
EC _cancer_ (µg/m^3^)	Max	19.285E-5	1.2847E-4	4.1873E-5	6.1291E-5	4.9403E-5	4.4779E-5	4.3089E-4
	Mean	8.8400E-5	1.2800E-4	4.1800E-5	6.0900E-5	4.9200E-5	4.4700E-5	4.3000E-4
	Min	3.1253E-5	1.2829E-4	4.1662E-5	6.0657E-5	4.9059E-5	4.4594E-5	4.2904E-4
ELCR	Max	3.654E-8	1.336E-8	4.354E-9	6.374E-9	5.137E-9	4.657E-9	5.601E-9
	Mean	1.250E-8	1.330E-8	4.340E-9	5.280E-9	5.120E-9	4.650E-9	5.600E-9
	Min	0.528E-8	1.334E-8	4.332E-9	6.308E-9	5.110E-9	4.637E-9	5.577E-9
BAD (mg)	Max	24.948	16.6320	5.4222	7.9296	6.3504	5.7960	6.9720
	Mean	8.7556	16.6143	5.4062	7.8869	6.3739	5.7865	6.9678
	Min	0.6552	16.5984	5.3928	7.8540	6.3966	5.7750	6.9636
PBL (mg/L)	Max	0.1520	0.1078	0.0034	0.0623	0.0049	0.0044	0.0045
	Mean	0.0540	0.0110	0.0044	0.0030	0.0240	0.0043	0.0043
	Min	0.0043	0.0095	0.0026	0.0053	0.0037	0.0039	0.0040

EC: Exposure Concentration, HQ: Hazard Quotient, ELCR: Excessive Lifetime Cancer Risk, BAD: Biological Absorbed Dose, PBL: Peak Blood Level.

**Table 2:** Risk calculations of health hazard assessment based on area formaldehyde exposure in laboratory workplaces.

Personal Risk Assessment		Sampling Sites						
		Pathology	Bacteriology	Hematology	Biochemistry	Serology	Reception	Services room
EC _noncancer_ (µg/m^3^)	Max	0.0065	0.0043	0.0018	0.0023	0.0021	0.0020	0.0047
	Mean	0.0023	0.0043	0.0019	0.0022	0.0022	0.0021	0.0044
	Min	0.0001	0.0043	0.0018	0.0021	0.0021	0.0020	0.0041
HQ	Max	0.1664	0.1100	0.0475	0.0607	0.0551	0.0531	0.1195
	Mean	0.0580	0.1100	0.0474	0.0560	0.0550	0.0530	0.1121
	Min	0.0020	0.1095	0.0474	0.0550	0.0549	0.0529	0.1047
EC _cancer_ (µg/m^3^)	Max	28.109E-4	1.858E-3	8.228E-4	10.250E-4	9.293E-4	8.946E-4	2.019E-3
	Mean	9.860E-4	1.900E-3	8.010E-4	9.500E-4	9.300E-4	8.900E-4	1.890E-3
	Min	0.738E-4	1.856E-3	8.003E-4	9.290E-4	9.271E-4	8.942E-4	1.767E-3
ELCR	Max	8.242E-9	2.416E-8	1.042E-8	1.332E-8	1.208E-8	1.164E-8	2.624E-8
	Mean	3.390E-9	2.410E-8	1.040E-8	1.230E-8	1.210E-8	1.160E-8	2.460E-8
	Min	0.959E-9	2.413E-8	1.040E-8	1.208E-8	1.205E-8	1.162E-8	2.298E-8
BAD (mg)	Max	45.402	30.072	12.982	15.111	15.048	14.490	32.676
	Mean	15.934	30.054	12.966	15.069	15.025	14.480	32.671
	Min	1.1970	30.038	12.952	15.036	15.002	14.469	32.667
PBL (mg/L)	Max	0.2780	0.2525	0.0083	0.1188	0.1002	0.1121	0.0214
	Mean	0.0990	0.0201	0.0073	0.0622	0.0284	0.0110	0.0190
	Min	0.0099	0.1724	0.0063	0.0102	0.0087	0.0097	0.0165

EC: Exposure Concentration, HQ: Hazard Quotient, ELCR: Excessive Lifetime Cancer Risk, BAD: Biological Absorbed Dose, PBL: Peak Blood Level.

### Cancer and non-cancer risk assessment

The calculated Excess Cancer Risk (ELCR) for all the sampling sites was significantly lower than the acceptable carcinogenic risk limit (ELCR=10^-4^). Similarly, the Hazard Quotient (HQ) values for all samples were below the reference level (HQ = 1), indicating a negligible risk of non-carcinogenic effects. These findings suggest that the exposure levels measured by the study method were unlikely to cause severe adverse health outcomes (Table 1, Table 2).

### Respiratory symptoms

**Fig. 6:**
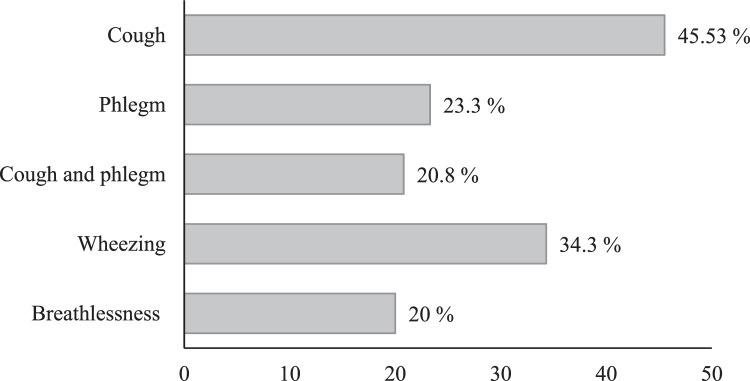
Frequency of respiratory symptoms among medical laboratory staff exposed to formaldehyde.

Fig. 6 shows a high prevalence of cough followed by wheezing, phlegm, and breathlessness among the participants across all sampling sites. The observed variations, with departments like pathology and bacteriology exhibiting higher prevalence of cough, phlegm, wheeze, and breathlessness, highlight the value of analyzing self-reported health data along environmental measurements.

The data presented in Table 3 provides insights into the prevalence of self-reported respiratory irritation among staff in various laboratory departments. Throat irritation was the most frequent symptom across all departments, affecting over 27 % of staff in each location. This finding is consistent with respiratory irritation, as this is a common symptom associated with formaldehyde exposure [24,25]. This consistency strengthens the validity of the self-reported data.

**Table 3:** Self-reported irritation by hospital laboratory staff exposed to formaldehyde.

Sampling sites	Respiratory irritation ( %)			
	Eye	Oral	Nasal	Throat
Pathology	13.66	21.66	13.00	28.33
Biochemistry	22.50	23.33	24.16	36.33
Bacteriology	18.20	22.20	15.80	27.40
Serology	21.80	23.20	23.80	35.60
Hematology	23.00	23.20	25.40	36.40
Receptionist	22.50	23.25	25.75	35.75
Attendant	18.50	23.00	16.50	30.50

Furthermore, the data revealed variations in the frequency of other symptoms between departments. For example, pathology and haematology had slightly higher percentages for eye and oral irritation compared to other areas. Reception and service staff reported lower percentages for all irritation symptoms compared to laboratory personnel. The observed variations in irritation prevalence across departments suggest a potential link between specific work areas and the likelihood of experiencing these symptoms. This aligns with the possibility of higher exposure risks in certain laboratory sections.

The high prevalence of respiratory symptoms among participants, particularly in the pathology and bacteriology departments can be attributed to several key factors related to formaldehyde exposure. First, our measurements indicate that these departments have significantly higher concentrations of airborne formaldehyde compared to other areas. Formaldehyde is a potent respiratory irritant known to cause inflammation and irritation of the respiratory tract, leading to symptoms like coughing, wheezing, and phlegm production [1]. The elevated levels of formaldehyde in these departments are a primary contributor to the increased prevalence of these respiratory symptoms.

The frequent use of formaldehyde for specimen preservation, tissue fixation, and disinfection in pathology and bacteriology departments results in continuous release of formaldehyde vapors into the air. This increases the exposure risk for workers, respiratory symptoms and potentially may lead to chronic respiratory issues.

Ineffective local exhaust ventilation (LEV) and general ventilation systems cause formaldehyde vapor to accumulate in the air, increasing respiratory exposure for workers.

Moreover, workers in these departments often work in close proximity to formaldehyde sources such as storage containers, preparation areas, and dissection tables. This close proximity increases their direct exposure risk, as tasks like tissue fixation and specimen preparation release significant amounts of formaldehyde, directly impacting the respiratory health of nearby workers. Improper storage of formaldehyde and inadequate disposal methods can lead to increased exposure.

Finally, specific work practices including the handling and transferring of formaldehyde solutions, contribute to the release of formaldehyde vapours. Inadequate use of personal protective equipment (PPE) and improper handling techniques further increase the exposure risk and exacerbate respiratory symptoms among workers.

Overall, the data on respiratory symptoms across various laboratory sections strengthens the method's ability to assess potential health risks associated with exposure. The method incorporated assessments of respiratory irritants to examine a potential link between exposure levels and the observed variations in symptom prevalence. This comprehensive approach, encompassing both environmental monitoring and human health data, strengthens the method's validity in identifying potential health risks in laboratory settings.

Table 4 presents a comparison of lung function parameters measured through pulmonary function tests among participants from different laboratory sections. While some lung function measures, like FVC (Forced Vital Capacity) and FEV_1_ (Forced Expiratory Volume in 1 second), showed no statistically significant differences (p-value > 0.05) across most departments, others like PEFR (Peak Expiratory Flow Rate) did exhibit significant variations (p-value < 0.05). Pulmonary function test data provided objective measurements of lung function, complementing the self-reported information on respiratory symptoms.

**Table 4:** The comparison of the pulmonary function tests of participants in medical laboratories.

Respiratory functional (Mean ± SD)								
Characteristics	Pathology	Bacteriology	Hematology	Biochemistry	Serology	Reception	Services room	*p-value*
FVC (L)	3.36 ± 0.11	4.01 ± 0.51	3.53 ± 0.53	2.58 ± 0.47	3.64 ± 0.52	4.73 ± 0.84	4.84 ± 0.01	0.150
FEV_1_ (L)	2.94 ± 0.14	3.09 ± 0.47	2.94 ± 0.25	2.50 ± 0.44	3.08 ± 0.68	4.11 ± 0.73	4.66 ± 0.01	0.701
FEV_1_ %	87.63 ± 1.22	76.42 ± 3.60	83.86 ± 1.75	81.15 ± 10.17	80.36 ± 7.40	86.80 ± 0.14	96.30 ± 0.01	0.096
PEFR (L/s)	4.65 ± 0.66	6.70 ± 0.84	3.39 ± 0.19	3.27 ± 0.87	4.62 ± 1.24	7.38 ± 1.19	7.01 ± 0.01	0.016*
FEF _25 % -75 %_ (L/s)	3.44 ± 0.10	3.01 ± 0.68	3.03 ± 0.22	2.61 ± 0.64	3.82 ± 1.10	4.55 ± 0.65	6.42 ± 0.01	0.091

*P-Value≤0.05, FVC: Forced Vital Capacity, FEV_1_: Forced Expiratory Volume in 1 second, PEF: Peak Expiratory Flow Rate, FEF: forced expiratory flow.

The pathology department showed the lowest average values for several lung function measures (FVC, FEV_1_, and PEFR), although the statistical significance varies. This finding indicates further investigation. The lung function parameters of reception and service staff, presumably not involved in direct chemical handling, appear to be within the normal range and serve as a reference point for comparison with other departments. Pulmonary function abnormalities can indicate early signs of lung function decline before symptoms even arise. This allows for preventive measures to be implemented before significant health problems develop. The observed variations in lung function measures across departments can help target further investigations and interventions in areas with potentially higher exposure risks, like pathology in this case. This risk assessment method considered both objective and subjective data to offer a more comprehensive approach to evaluating respiratory health risks in medical laboratory work settings.

### Statistical significance

The Kruskal-Walli's test results showed a statistically significant relationship between the estimated PBL (blood formaldehyde levels), cancer and non-cancer risks with the sampling sites in both personal and area samples (P-value< 0.05). This statistically significant relationship strengthens the validity of the study method as it demonstrates that the observed differences in exposure levels were not random.

**Limitations**

Personal measurements provided data results about an individual's potential exposure while area measurements pointed out a general idea of airborne formaldehyde levels within a particular workspace. This comprehensive approach strengthened the method's ability to identify potential risk areas. By combining personal and area measurements and comparing them to a standard limit, the method offered valuable data. However, future studies with a larger sample size and potentially incorporating personal monitoring over a longer duration could provide an even more robust validation of the method.

The study employed a convenience sampling approach, inviting all staff in the 74 laboratories to participate. This method might not be representative of the entire laboratory workforce within the hospitals. Workers who are less concerned about exposure or have different work patterns might be less likely to participate. Applying a random sampling method would provide a more generalizable picture of the laboratory workforce.

While this study provided valuable data on formaldehyde exposure levels in laboratory settings through short-term measurements, several limitations must be acknowledged. Short-term exposure data estimate exposure levels over brief periods which may not fully represent the variations in exposure that occur over longer periods. This limitation can lead to either underestimation or overestimation of true exposure levels. Furthermore, short-term data do not provide sufficient information to assess the health risks associated with chronic exposure, such as cancer, which typically result from prolonged exposure to formaldehyde.

Formaldehyde levels can vary significantly based on daily workloads, specific activities, and seasonal changes, and short-term measurements might not consider these variations, leading to incomplete assessments of exposure. Thus, short-term assessments might reflect conditions during a specific sampling period and may not account for unusual events that could significantly impact exposure levels.

To gain a more accurate understanding of fluctuating exposure levels and associated health risks, longer-term studies are essential. These studies can provide comprehensive exposure profiles, assess chronic health risks, identify patterns and trends, and evaluate the effectiveness of mitigation measures over time. Future assessments incorporating well-designed local and general exhaust ventilation systems and extended monitoring periods will be crucial in mitigating health hazards and ensuring sustained protection of worker health. Thus, conducting the risk assessment over a longer period can examine variations in exposure and health outcomes over time.

Future assessments should incorporate the installation and evaluation of well-designed local and general exhaust ventilation systems to determine their effectiveness in mitigating formaldehyde exposure in hospital laboratories. Implementing these ventilation improvements could provide more data on the reduction of airborne formaldehyde exposure concentrations and the corresponding decrease in health risks for laboratory personnel. This approach would enable a more accurate assessment of the overall efficacy of ventilation interventions and contribute to the development of best practices for maintaining safe work environments in medical laboratories.

Additionally, longitudinal studies that monitor airborne formaldehyde exposure levels and health outcomes over time, before and after the implementation of ventilation improvements, would provide valuable insights into the long-term benefits and sustainability of these interventions.

The data establishes a correlation between working in certain laboratory areas and experiencing respiratory symptoms. However, it cannot definitively prove that these symptoms are caused by exposure to substances in those workplaces. Also, lung function can vary naturally between individuals due to factors like age, height, and sex. The data needs to be interpreted in the context of individual characteristics. Furthermore, some pulmonary function test abnormalities may reflect temporary changes in lung function rather than permanent damage. Further monitoring might be needed to assess the persistence of these changes.

Finally, because the data relies on self-reported symptoms, participants' memory and interpretation can influence their responses. To enhance the reliability and accuracy of these reports, several measures can be implemented. In the study, standardized questionnaires were used to ensure consistent interpretation of questions and reduce variability in responses. Further risk assessment studies should increase the frequency of data collection to reduce recall bias. Also, training participants to accurately recognize and report symptoms can help. Using mobile apps for real-time symptom reporting, can further improve data accuracy.

## CRediT authorship contribution statement

**Marzieh Belji Kangarlou:** Project administration, Conceptualization, Methodology, Investigation, Validation, Writing – original draft, Writing – review & editing. **Alireza Dehdashti:** Supervision, Funding acquisition, Project administration, Resources, Conceptualization, Methodology, Writing – original draft, Writing – review & editing. **Elaheh Saleh:** Formal analysis, Data curation.

## Declaration of competing interest

The authors declare that they have no known competing financial interests or personal relationships that could have appeared to influence the work reported in this paper.

## Data Availability

Data will be made available on request.
